# NF-κB promotes leaky expression of adenovirus genes in a replication-incompetent adenovirus vector

**DOI:** 10.1038/srep19922

**Published:** 2016-01-27

**Authors:** M. Machitani, F. Sakurai, K. Wakabayashi, K. Nakatani, K. Shimizu, M. Tachibana, H. Mizuguchi

**Affiliations:** 1 Laboratory of Biochemistry and Molecular Biology, Graduate School of Pharmaceutical Sciences, Osaka University, 1-6 Yamadaoka, Suita, Osaka 565-0871, Japan; 2 Laboratory of Regulatory Sciences for Oligonucleotide Therapeutics, Clinical Drug Development Unit, Graduate School of Pharmaceutical Sciences, Osaka University, 1-6 Yamadaoka, Suita, Osaka, 565-0871, Japan; 3 Faculty of Pharmacy, Osaka Ohtani University, 3-11-1 Nishikiorikita, Tondabayashi, Osaka, 584-8540, Japan; 4 Laboratory of Hepatocyte Regulation, National Institutes of Biomedical Innovation, Health and Nutrition, 7-6-8 Saito, Asagi, Ibaraki, Osaka 567-0085, Japan; 5 iPS Cell-Based Research Project on Hepatic Toxicity and Metabolism, Graduate School of Pharmaceutical Sciences, Osaka University, 1-6 Yamadaoka, Suita, Osaka 565-0871, Japan; 6 Global Center for Advanced Medical Engineering and Informatics, Osaka University, 2-2 Yamadaoka, Suita, Osaka 565-0871, Japan; 7 Graduate School of Medicine, Osaka University, 2-2 Yamadaoka, Suita, Osaka 565-0871, Japan

## Abstract

The replication-incompetent adenovirus (Ad) vector is one of the most promising vectors for gene therapy; however, systemic administration of Ad vectors results in severe hepatotoxicities, partly due to the leaky expression of Ad genes in the liver. Here we show that nuclear factor-kappa B (NF-κB) mediates the leaky expression of Ad genes from the Ad vector genome, and that the inhibition of NF-κB leads to the suppression of Ad gene expression and hepatotoxicities following transduction with Ad vectors. Activation of NF-κB by recombinant tumor necrosis factor (TNF)-α significantly enhanced the leaky expression of Ad genes. More than 50% suppression of the Ad gene expression was found by inhibitors of NF-κB signaling and siRNA-mediated knockdown of NF-κB. Similar results were found when cells were infected with wild-type Ad. Compared with a conventional Ad vector, an Ad vector expressing a dominant-negative IκBα (Adv-CADNIκBα), which is a negative regulator of NF-κB, mediated approximately 70% suppression of the leaky expression of Ad genes in the liver. Adv-CADNIκBα did not induce apparent hepatotoxicities. These results indicate that inhibition of NF-κB leads to suppression of Ad vector-mediated tissue damages *via* not only suppression of inflammatory responses but also reduction in the leaky expression of Ad genes.

The replication-incompetent E1-deleted adenovirus (Ad) vector is the most promising vector for both gene therapy and basic studies due to its advantages as a gene delivery vehicle, which include high titer production and high transduction efficiencies in both dividing and non-dividing cells^1^,^2^. In a conventional Ad vector, the E1 (E1A,E1B) gene is replaced by a transgene expression cassette and the Ad vector is propagated in E1-expressing cell lines, such as HEK293 cells. The E1A gene is the first transcription unit to be expressed immediately after wild-type Ad infection, and transactivates the expression of other Ad genes. Thus, no Ad genes should be expressed from the Ad vector genome due to the lack of the E1A and E1B genes in the Ad vector genome. However, expression of Ad genes, including the E2, E4 and pIX genes, is indeed detected in the Ad vector-transduced cells, indicating that the Ad genes are expressed from the Ad vector genome in an E1A gene-independent manner^3^,^4^,^5^,^6^. Further, the leaky expression results in immune responses against Ad proteins and Ad protein-induced cellular toxicity, leading to tissue damages such as hepatotoxicities^7^,^8^,^9^.

Various types of next-generation Ad vectors which can overcome these disadvantages have been developed^3^,^4^,^8^. For example, an Ad vector lacking the E2A gene expression achieved a reduction in the immune responses and the prolonged transgene expression profiles^10^. A helper-dependent Ad (HD-Ad) vector, in which all viral encoding genes were deleted, also showed a reduction in the production of inflammatory cytokines and the tissue damages^11^,^12^,^13^. In addition, we recently developed an Ad vector that suppresses the leaky expression of Ad genes by inserting microRNA-targeted sequences in the Ad vector genome^9^. Nonetheless, problems remain with all of these novel Ad vectors, including the complexity of the Ad vector preparation and some remaining Ad vector-induced hepatotoxicities. To overcome these drawbacks, the mechanisms of the E1A gene-independent leaky expression of Ad genes should be clarified.

Transduction with Ad vectors causes activation of a number of transcriptional factors. For example, injection with recombinant Ad vectors immediately induces production of inflammatory cytokines associated with innate immune responses^14^,^15^, suggesting that several transcriptional factors would be activated in the innate immune responses. In this study, among these transcriptional factors, we focused on nuclear factor-kappa B (NF-κB) because NF-κB has been demonstrated to play a crucial role in the expression of numerous viral genes^16^. NF-κB is a ubiquitous transcriptional factor which promotes the expression of a large number of genes, particularly those from gene families associated with host immune responses^16^. Thus, NF-κB plays a crucial role in immune responses. Although NF-κB functions as a homodimer or heterodimer (p50/p50, p50/p65, and p52/RelB), canonical NF-κB is a heterodimer which is composed of p50 and p65. Under normal conditions, NF-κB stays in the cytoplasm *via* association with IκBα, an NF-κB-inhibitory factor. Upon the stimulation by cytokines and pathogens, IκBα is phosphorylated and degraded, leading to localization of NF-κB into the nucleus from the cytoplasm. In the nucleus, translocated NF-κB promotes the expression of target genes by binding to the consensus sequence (5′GGGACTTTCC-3′) in their promoter region.

In the present study, we have demonstrated that NF-κB promotes Ad gene expression following transduction with an Ad vector and a wild-type Ad (WT-Ad). Further, an Ad vector expressing a dominant-negative IκBα (Adv-CADNIκBα), which is a negative regulator of NF-κB, mediated the significant suppression of the leaky expression of Ad genes, with the result that no apparent hepatotoxicities were induced.

## Materials and Methods

### Cell lines and animals

HEK293 (a transformed embryonic kidney cell line) and HeLa (a human epithelial carcinoma cell line) cells were cultured in Dulbecco’s modified Eagle’s medium supplemented with 10% fetal bovine serum (FBS), streptomycin (100 μg/ml), and penicillin (100 U/ml). H1299 (a non-small cell lung carcinoma cell line) cells were cultured in RPMI1640 supplemented with 10% FBS, streptomycin (100 μg/ml), and penicillin (100 U/ml). Female C57BL/6 mice aged 5–6 weeks were obtained from Nippon SLC (Shizuoka, Japan). Rag2/Il2rγ double knockout mice of C57BL/6 background, aged 5–7 weeks, were obtained from Taconic Farms (Hudson, NY)^17^,^18^. All mouse experimental procedures used in this study were approved by Animal Experimentation Committee of Osaka University and performed in accordance with institutional guidelines for animal experiments at Osaka University.

### Reagents

Recombinant human and mouse tumor necrosis factor (TNF)-α were purchased from Invivogen (San Diego, CA) and Peprotech (Rocky Hill, NJ), respectively. NF-κB inhibitors, BAY11-7082 and MG-132, were purchased from Invivogen. Recombinant human interferon-α (IFN-α) was purchased from PBL interferon source (Piscataway, NJ).

### Plasmids

pNF-κB-Luc, which contains the firefly luciferase (FLuc) gene expression cassette driven by a promoter containing a consensus sequence for the NF-κB binding, was purchased from Agilent Technologies (Santa Clara, CA). pHMCMV-RLuc, a reporter plasmid carrying a cytomegalovirus (CMV) promoter-driven renilla luciferase (RLuc) expression cassette, was constructed previously^19^.

**Figure 1:**
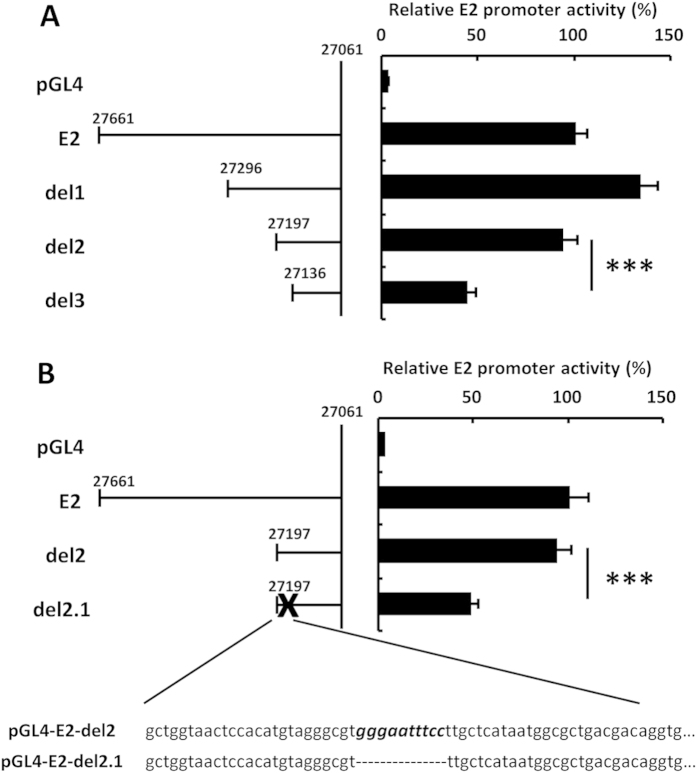
Reduction in the E2 promoter activity by deletion of an NF-κB binding site. (**A**,**B**) HeLa cells were co-transfected with pHMCMV6-RLuc and reporter plasmids carrying the E2-promoter-driven FLuc expression cassette (pGL4, pGL4-E2, pGL4-E2-del1, pGL4-E2-del2, pGL4-E2-del2.1, or pGL4-E2-del3). After a 24-h incubation, luciferase activity was determined. The data show FLuc activity normalized by RLuc activity in the cells. Schematic diagrams of each promoter are shown at the left of the graph. (B) Nucleotide sequence around the NF-κB binding site (italic type) in the E2 promoter region. The NF-κB binding sequence is deleted in pGL4-E2-del2.1. These data are expressed as the means ± S.D. (n = 4). ***p < 0.001.

The reporter plasmids pGL4-E1A, -E1B, -E2, -E3, and -E4, which have the promoter sequences of the respective Ad genes upstream of the FLuc gene, were constructed as follows: the fragment containing the E2 promoter sequence (bp 27061–27661) was amplified by PCR using the primers E2p-F and E2p-R, and ligated with a *Hind*III/*Xho*I fragment of pGL4.10 (Promega, Madison, WI), resulting in pGL4-E2. pGL4-E1A, -E1B, -E3, and -E4, which contain the E1A (bp 1–546), E1B (bp 1336–1702), E3 (bp 26987–27578), and E4 (bp 35530–35939) promoter region, respectively, were similarly constructed using the corresponding primers. The mutated reporter plasmids pGL4-E2-del1, -del2, and -del3, which have shortened E2 promoter sequences (bp 27061–27296, 27061–27197, and 27061–27136, respectively) upstream of the FLuc gene, were constructed as follows. First, pGL4-E2 was digested with *Eco*RV/*Xho*I. The resulting fragments were then self-ligated after the sticky end was converted to a blunt end, resulting in pGL4-E2-del1. The fragment containing the shortened E2 promoter sequence (bp 27061–27197) was amplified by PCR using the primers E2-del2-F and E2-del2-R, and ligated with a *Hind*III/*Xho*I fragment of pGL4.10, resulting in pGL4-E2-del2. An *Afl*II/*Xho*I fragment of pGL4-E2 was ligated with the oligonucleotides, E2-del3-S and E2-del3-AS, which encode the shortened E2 promoter sequence (bp 27061–27136), resulting in pGL4-E2-del3. A reporter plasmid, pGL4-del2.1, which has the shortened E2 promoter sequences (bp 27061–27197) and lacks the predicted NF-κB binding site in the E2 promoter sequence, was constructed as follows: the predicted NF-κB binding site was mutated by using a QuikChange Site-Directed Mutagenesis Kit (Agilent Technologies) and the primers E2-del2.1-F and E2-del2.1-R, resulting in pGL4-E2-del2.1. The mutated sequence of pGL4-E2-del2.1 is shown in Fig. 1B. Sequences of the primers and oligonucleotides are shown in Table S1.

pAdHM4-CMV-Luc, the Ad vector plasmid carrying a CMV promoter-driven luciferase expression cassette, was constructed previously^9^. pAdHM4-CA-Luc and -DNIκBα, the Ad vector plasmid carrying a CA promoter (a β-actin promoter/CMV enhancer with a β-actin intron)-driven luciferase and dominant-negative IκBα gene expression cassette, respectively, were constructed as follows: pHMCA5^20^ was digested with *Not*I/*Xba*I, and ligated with the *Not*I/*Xba*I fragment of pCMVL1^9^, resulting in pHMCA5-Luc. The fragment containing the dominant-negative IκBα gene was amplified by PCR using pCMX-IκBαM (Addgene plasmid 12329; Addgene, Cambridge, MA), which contains the dominant-negative IκBα gene expression cassette, and the primers DNIκBα-F and DNIκBα-R, and ligated with the *Not*I/*Xba*I fragment of pHMCA5, resulting in pHMCA5-DNIκBα. pHMCA5-Luc and -DNIκBα were digested with I-*Ceu*I/PI-*Sce*I, and then ligated with I-*Ceu*I/PI-*Sce*I−digested pAdHM4^21^, resulting in pAdHM4-CA-Luc and pAdHM4-CA-DNIκBα, respectively.

### Viruses

WT-Ad (human Ad serotype 5) was obtained from the American Type Culture Collection (ATCC). Recombinant Ad vectors were prepared by an *in vitro* improved ligation method as follows^22^,^23^; all Ad vector plasmids used in this study have *Pac*I-flanked Ad vector genome. Ad vector plasmids, pAdHM4-CMV-Luc, pAdHM4-CA-Luc, and pAdHM4-CA-DNIκBα, were digested with *Pac*I to release the recombinant Ad vector genome, and were each transfected into HEK293 cells using Lipofectamine 2000 (Life Technologies, Carlsbad, CA), resulting in Adv-CMVLuc, Adv-CALuc, and Adv-CADNIκBα, respectively. Adv-CALacZ, an Ad vector expressing LacZ, was constructed previously^24^. These Ads were amplified and purified by two rounds of cesium chloride-gradient ultracentrifugation, dialyzed, and stored at −80 °C^22^. The virus particles (VP) were determined by a spectrophotometrical method^22^. Determination of infectious units (IFU) was accomplished using an Adeno-X Rapid Titer Kit (Clontech, Mountain View, CA).

### Prediction of NF-κB binding sites in the Ad genome

Transcriptional factors binding to the E2 promoter region and the putative binding sites in the promoter region were predicted using TFSEARCH (http://www.cbrc.jp/research/db/TFSEARCH.html).

### Analysis of the promoter activities using reporter plasmids

Cells were seeded in a 24-well plate at a density of 5 × 10^4^ cells/well. On the following day, cells were co-transfected with FLuc-expressing reporter plasmids (500 ng/ml) (constructed as shown above) and pHMCMV-RLuc (160 ng/ml) using Lipofectamine 2000 (Life Technologies). Following a 6-h incubation, the medium was changed. Luciferase activity in cells was determined using the Dual Luciferase Reporter Assay System (Promega) 24 h after transfection.

For evaluation of the effects of TNF-α on the promoter activities, cells were treated with TNF-α (100 ng/ml) 24 h after transfection with plasmids as described above. Luciferase assay was performed 24 h after TNF-α treatment.

### Quantitative RT-PCR analysis of Ad gene expression

Cells were seeded in a 24-well plate at a density of 5 × 10^4^ cells/well. On the following day, WT-Ad and Ad vectors were added to the cells at the indicated titers. Following the indicated incubation periods, total RNA was isolated from cells using ISOGEN (Nippon Gene, Tokyo, Japan). cDNA was synthesized using 500 ng of total RNA with a Superscript VILO cDNA synthesis kit (Life Technologies). Real-time RT-PCR analysis was performed using Fast SYBR Green Master Mix (Life Technologies) and StepOnePlus real-time PCR systems (Life Technologies). Sequences of the primers used in this study are described in Table S1.

For evaluation of the effects of TNF-α on Ad gene expression, cells were pre-treated with TNF-α at a concentration of 100 ng/ml for 5 h, followed by incubation with WT-Ad and Ad vectors. Total RNA was recovered for quantitative RT-PCR analysis 12 h after addition of WT-Ad and Ad vectors.

For inhibition of NF-κB, cells were pre-treated with BAY11-7082 and MG-132 at 10 μM and 2.5 μM, respectively, for 1 h, followed by incubation with WT-Ad and Ad vectors. Total RNA was recovered 12 h after the addition of WT-Ad and Ad vectors.

For knockdown of p50, cells were transfected with an siRNA against p50 (sip50) (Gene Design, Osaka, Japan) using Lipofectamine 2000 (Life Technologies) according to the manufacturer’s instructions. The target sequence of sip50 was 5′-aaggggctataatcctggact-3′. Control siRNA was purchased from Qiagen (Allstars Negative Control siRNA; Qiagen, Hilden, Germany). Cells were treated with WT-Ad and Ad vectors 48 h after siRNA transfection. Total RNA was recovered 12 h after addition of WT-Ad and Ad vectors.

### Western blotting analysis

Western blotting assay was performed as previously described^13^. Briefly, whole-cell extracts were prepared and electrophoresed on 10% sodium dodecyl sulfate (SDS)-polyacrylamide gels under reducing conditions, followed by electrotransfer to PVDF membranes (Millipore, Bedford, MA). After blocking with 5% skim milk prepared in TBS-T (tween-20, 0.1%), the membrane was incubated with a mouse anti-E1A antibody (clone name) (Abcam, Cambridge, UK), a rabbit anti-p50 antibody (H-119) (Santa Cruz Biotechnology, Santa Cruz, CA), or mouse anti-β-actin (Sigma-Aldrich, St. Louis, MO), followed by incubation in the presence of horseradish peroxidase (HRP)-labeled anti-rabbit or anti-mouse IgG antibody (Cell Signaling Technology, Danvers, MA). The intensity of protein bands was quantified by Image J software.

### Determinaton of Ad genome copy numbers

Cells were treated with WT-Ad and Ad vectors in a manner similar to that described above. Following the indicated incubation periods, total DNA, including Ad genomic DNA, was isolated from the cells infected with Ads using a DNeasy Blood & Tissue Kit (Qiagen). After isolation, Ad genome copy numbers were quantified using a StepOnePlus real-time PCR system (Life Technologies) as previously described^25^. Sequences of the primers and probes used in this study are described in Table S1.

### Chromatin immunoprecipitation (ChIP) assay

HeLa cells were infected with WT-Ad at 100 VP/cell. Following a 24-h incubation, cells were treated with formaldehyde at a final concentration of 1% for crosslinking, and then genomic DNA was fragmented by sonication. The DNA fragment-protein complexes were immunoprecipitated using a rabbit anti-p65 antibody (ab7970) (Abcam) or normal rabbit IgG (Cell Signaling). The ChIP assay kit was purchased from Merck Millipore (Darmstadt, Germany). The precipitated DNA copy numbers were determined by quantitative PCR using the primers shown in Table S1.

### *In vivo* Ad gene expression analysis

Ad vectors were intravenously administered to mice at a dose of 10^9^ IFU/mouse *via* a tail vein. Total RNA was extracted from the livers 48 h after administration, and the Ad gene mRNA levels were determined by quantitative RT-PCR analysis.

### Analysis of Ad vector-mediated hepatotoxicities

Ad vectors were intravenously administered to mice at a dose of 10^9^ IFU/mouse *via* a tail vein. The blood samples were collected *via* retro-orbital bleeding at the indicated days, and the serum samples were obtained after centrifugation. The serum alanine aminotransferase (ALT) and aspartate aminotransferase (AST) levels were determined using a transaminase-CII-test kit (Wako, Osaka, Japan).

### Statistical analysis

Statistical significance was determined using Student’s *t*-test. Data are presented as the means ± S.D or S.E.

## Results

### The NF-κB binding site is essential for the E2 promoter activity

In order to identify the transcriptional factors crucial for leaky expression of the Ad genes, functional analysis of a promoter of the E2 gene, which shows leaky expression from the Ad vector genome^5^, was performed using reporter plasmids (Fig. 1A). The luciferase expression levels from pGL4-E2-del3, which has the shortened promoter sequence (bp 27061–27136) of the E2 gene upstream of the FLuc gene, was significantly lower than that from pGL4-E2, which has the promoter sequence (bp 27061–27661) of the E2 gene upstream of the FLuc gene (Fig. 1A), suggesting that the sequence between bp 27136–27197 in the E2 promoter was significantly involved in the E2 promoter activity. Next, we performed *in silico* analysis using TFSEARCH, a program that searches for transcriptional factor binding sites, to identify transcriptional factor binding sites in the sequence between bp 27136–27197. This analysis revealed an NF-κB binding site (5′-gggaatttcc-3′) in this sequence (Fig. 1B). To examine whether the NF-κB binding site is essential for the E2 promoter activity, the NF-κB binding site in the E2 promoter sequence of pGL4-E2-del2 was deleted. The deletion of the NF-κB binding site resulted in a significant reduction in the luciferase activity of pGL4-E2-del2 (Fig. 1B). These results suggested that the NF-κB binding site was important for the E2 promoter activity.

### Activation of NF-κB promotes Ad gene expression and Ad replication

**Figure 2:**
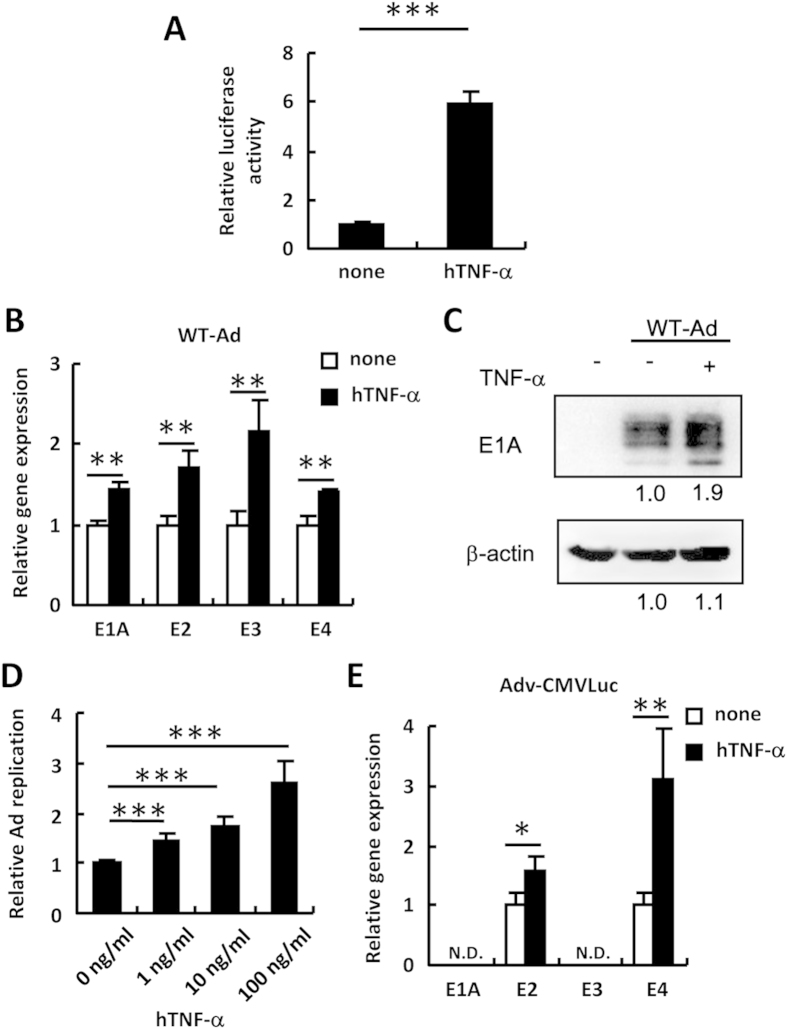
Promotion of Ad early gene expression and replication by TNF-α stimulation. (**A**) HeLa cells were transfected with pNF-κB-Luc, followed by treatment with recombinant hTNF-α at 100 ng/ml. After 24-h incubation, luciferase activity was determined. The data show FLuc activity normalized by RLuc activity. (**B**,**C**) HeLa cells were pre-treated with hTNF-α at 100 ng/ml for 5 h, followed by infection with WT-Ad at 100 VP/cell. After 12-h incubation, the E1A, E2, E3, and E4 mRNA levels in the cells were determined by quantitative RT-PCR (**B**). After 24-h incubation, the E1A protein levels in the cells were determined by western blotting analysis (**C**). The intensity of E1A expression was quantified using Image J software. (**D**) HeLa cells were pre-treated with hTNF-α at the indicated concentration for 5 h, followed by infection with WT-Ad at 100 VP/cell. After 24-h incubation, Ad genome copy numbers in the cells were determined by quantitative PCR. (**E**) HeLa cells were pre-treated with hTNF-α at 100 ng/ml for 5 h, followed by transduction with Adv-CMVLuc at 100 VP/cell. After 12-h incubation, Ad gene mRNA levels in the cells were similarly determined. These data are expressed as the means ± S.D. (n = 3–4). *p < 0.05, **p < 0.01, ***p < 0.001.

In order to examine whether NF-κB is involved in the leaky expression of Ad genes, including the E2 gene, HeLa cells were pre-treated with recombinant human TNF-α (hTNF-α), followed by infection with WT-Ad. The reporter assay using pNF-κB-Luc, which carries the luciferase gene expression cassette driven by a promoter containing a consensus sequence for NF-κB binding, showed that hTNF-α treatment significantly activated NF-κB signaling (Fig. 2A). Activation of the NF-κB signaling by hTNF-α significantly (1.4-2-fold) enhanced the mRNA levels of the Ad early genes, including the E1A, E2, E3, and E4 genes, in HeLa cells following infection with WT-Ad (Fig. 2B). The E1A protein levels were also elevated by TNF-α treatment in WT-Ad-infected HeLa cells (Fig. 2C). WT-Ad replicated more efficiently in HeLa cells pre-treated with hTNF-α in a dose-dependent manner (Fig. 2D). In addition, hTNF-α treatment significantly enhanced the leaky expression of the E2 and E4 genes in HeLa cells following transduction with Adv-CMVLuc, a replication-incompetent Ad vector expressing FLuc (Fig. 2E). These results suggested that the activation of NF-κB by TNF-α promoted Ad gene expression and Ad replication.

### Inhibition of NF-κB negatively regulates Ad gene expression and Ad replication

**Figure 3:**
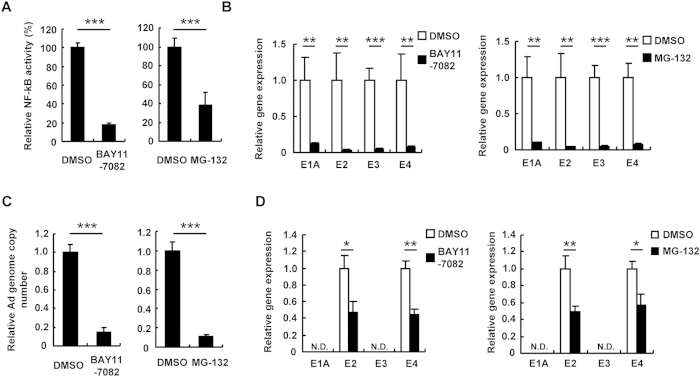
Suppression of Ad early gene expression and replication by NF-κB inhibitors. (**A**) HeLa cells were pre-treated with BAY11-7082 and MG-132 at 10 mM and 2.5 mM, respectively, for 1 h, followed by transfection with pNF-κB-Luc. After 24-h incubation, luciferase activity was determined. The data show FLuc activity normalized by RLuc activity. (**B**,**C**) HeLa cells were pre-treated with BAY11-7082 and MG-132 at 10 mM and 2.5 mM, respectively, for 1 h, followed by infection with WT-Ad at 100 VP/cell. After 12-h incubation, the E1A, E2, E3, and E4 mRNA levels in the cells were determined by quantitative RT-PCR (**B**). After 24-h incubation, Ad genome copy numbers in the cells were determined by quantitative PCR (**C**). (**D**) HeLa cells were pre-treated with BAY11-7082 and MG-132 at 10 mM and 2.5 mM, respectively, for 1 h, followed by infection with Adv-CMVLuc at 100 VP/cell. After 12-h incubation, Ad gene mRNA levels in the cells were similarly determined. These data are expressed as the means ± S.D. (n = 3–4). *p < 0.05, **p < 0.01, ***p < 0.001.

**Figure 4:**
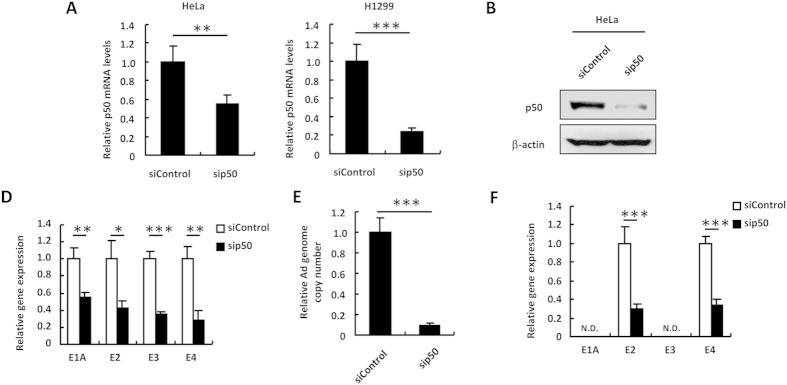
Suppression of Ad early gene expression and replication by NF-κB knockdown. (**A**,**B**) HeLa and H1299 cells were transfected with siControl or sip50. After 48-h incubation, mRNA and protein levels of p50 in the cells were determined by quantitative RT-PCR (**A**) and western blotting analysis (**B**), respectively. p50 mRNA levels in the cells transfected with siControl were normalized to 1. (**C**,**D**) HeLa cells were transfected with sip50 at 50 nM, followed by infection with WT-Ad at 100 VP/cell. After 12-h incubation, the E1A, E2, E3, and E4 mRNA levels in the cells were determined by quantitative RT-PCR (**C**). After 24-h incubation, Ad genome copy numbers in the cells were determined by quantitative PCR (**D**). (**E**) HeLa cells were transfected with sip50 at 50 nM, followed by transduction with Adv-CMVLuc at 100 VP/cell. After 12-h incubation, Ad gene mRNA levels in the cells were similarly determined. These data are expressed as the means ± S.D. (n = 3–4). *p < 0.05, **p < 0.01, ***p < 0.001.

Next, in order to examine the involvement of NF-κB in Ad gene expression and Ad replication, HeLa cells were pre-treated with the NF-κB inhibitors BAY11-7082 and MG-132, followed by infection with WT-Ad. The cell viabilities were not significantly reduced following treatment with BAY11-7082 and MG-132 (Figure S1). The reporter assay using pNF-κB-Luc showed that the treatment with BAY11-7082 and MG-132 largely inhibited NF-κB signaling (Fig. 3A). Inhibition of NF-κB signaling by BAY11-7082 and MG-132 resulted in more than 80% reduction in the E1A, E2, E3, and E4 gene expressions in WT-Ad-infected HeLa cells (Fig. 3B). Copy numbers of the WT-Ad genome were also suppressed by more than 80% in HeLa cells following treatment with BAY11-7082 and MG-132 (Fig. 3C). In addition, treatment with BAY11-7082 and MG-132 reduced the leaky expression of the E2 and E4 genes by approximately 50% in HeLa cells following transduction with Adv-CMVLuc (Fig. 3D). Next, in order to further examine the effects of NF-κB on Ad gene expression and Ad replication, p50, which is a component of NF-κB, was knocked down in HeLa cells. Transfection with sip50 resulted in a significant knockdown of p50 at both the mRNA and protein levels (Fig. 4A,B). The E1A, E2, E3, and E4 mRNA levels following infection with WT-Ad were suppressed by more than 50% in p50-knockdown cells, compared with those in control cells (Fig. 4C). An approximately 90% reduction in copy numbers of the WT-Ad genome was found in p50-knockdown HeLa cells (Fig. 4D). Knockdown of p50 also led to approximately 70% decreases in the leaky expression of the E2 and E4 genes following transduction with Adv-CMVLuc (Fig. 4E). Similar results were found in H1299 cells (Figure S2). These results indicated that NF-κB was essential for Ad gene expression and replication of WT-Ad, and that NF-κB induced the leaky expression of the Ad genes following transduction with Ad vectors.

### NF-κB binds to the E2 and E3 promoter

**Figure 5:**
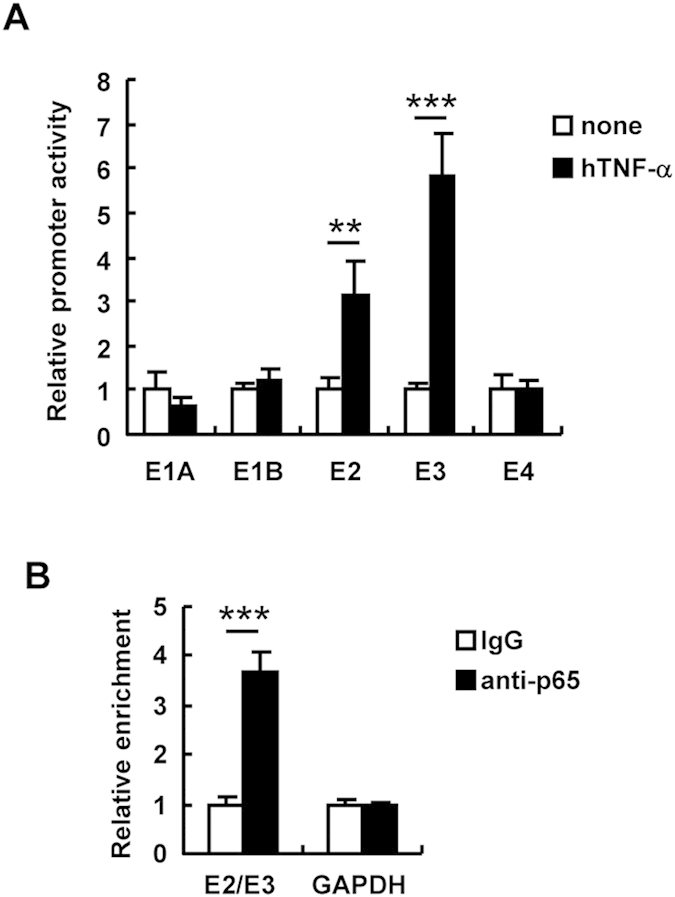
NF-κB-mediated enhancement of the E2 and E3 promoter activity. (**A**) HeLa cells were transfected with reporter plasmids carrying the expression cassette of Ad early gene promoter-driven FLuc (pGL4-E1A, -E1B, -E2, -E3, and -E4) for 24 h, followed by treatment with hTNF-α at 100 ng/ml. After 24-h incubation, luciferase activity was determined. (**B**) HeLa cells were infected with WT-Ad at 100 VP/cell. After 24-h incubation, NF-κB binding DNA levels were determined by ChIP assay. These data are expressed as the means ± S.D. (n = 3–4). **p < 0.01, ***p < 0.001.

In order to examine whether NF-κB enhances Ad gene promoter activities, HeLa cells were transfected with reporter plasmids carrying the Ad gene promoter-driven luciferase expression cassette, followed by treatment with hTNF-α. The E2 and E3 promoter activities, but not the activities of the E1A, E1B, or E4 promoter, were more than 3-fold enhanced by hTNF-α treatment (Fig. 5A). Next, we performed a ChIP assay using an antibody against p65, which is another component of NF-κB, to examine whether NF-κB directly binds to the E2 and E3 promoter region. The E2 and E3 promoter is a bidirectional promoter, which drives transcription of both the E2 and E3 genes. The ChIP assay demonstrated that NF-κB directly bound to the E2 and E3 promoter region (Fig. 5B), indicating that NF-κB activates the E2 and E3 gene expression *via* direct binding to the promoter region.

### IFN-α treatment suppresses Ad gene expression and Ad replication

Next, we investigated the involvement of other transcriptional factors in Ad gene expression. In order to activate transcriptional factors involved in innate immune responses, HeLa cells were pre-treated with recombinant human IFN-α, which activates STAT1, STAT2, and IRF9, followed by infection with WT-Ad. The reporter assay using pNF-κB-Luc showed that IFN-α treatment significantly inhibited NF-κB signaling (Figure S3A). Contrary to TNF-α treatment, IFN-α treatment significantly suppressed the mRNA levels of the Ad early genes, including the E1A, E2, E3, and E4 genes, in HeLa cells following infection with WT-Ad (Figure S3B). WT-Ad replicated less efficiently in HeLa cells pre-treated with IFN-α (Figure S3C). These results suggested that transcriptional factors activated by IFN-α did not promote Ad gene expression and Ad replication.

### NF-κB is involved in Ad gene expression in the mouse liver

**Figure 6:**
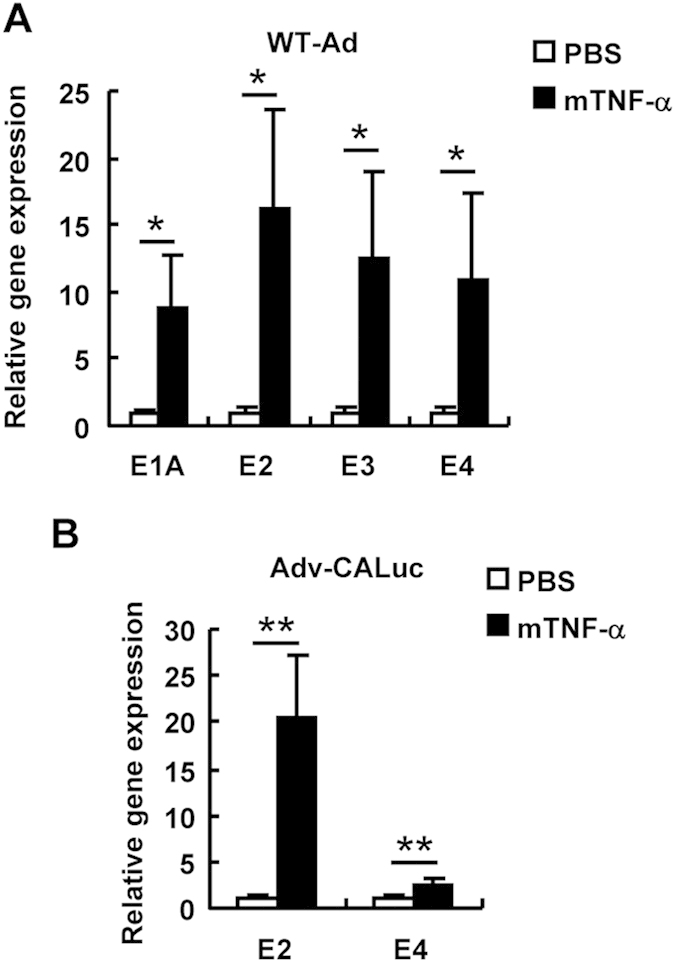
Promotion of Ad early gene expression by TNF-α stimulation in mouse liver. (**A**,**B**) C57BL/6 mice were intravenously administered 10^9^ IFU of WT-Ad (**A**) or Adv-CALuc (**B**), followed by intravenous administration of recombinant mTNF-α at 0.5 mg/mouse. Twenty-four hours after the administration, the E1A, E2, E3, and E4 mRNA levels in the mouse liver were determined by quantitative RT-PCR. These data are expressed as the means ± S.E. (n = 5–6). *p < 0.05, **p < 0.01, ***p < 0.001.

**Figure 7:**
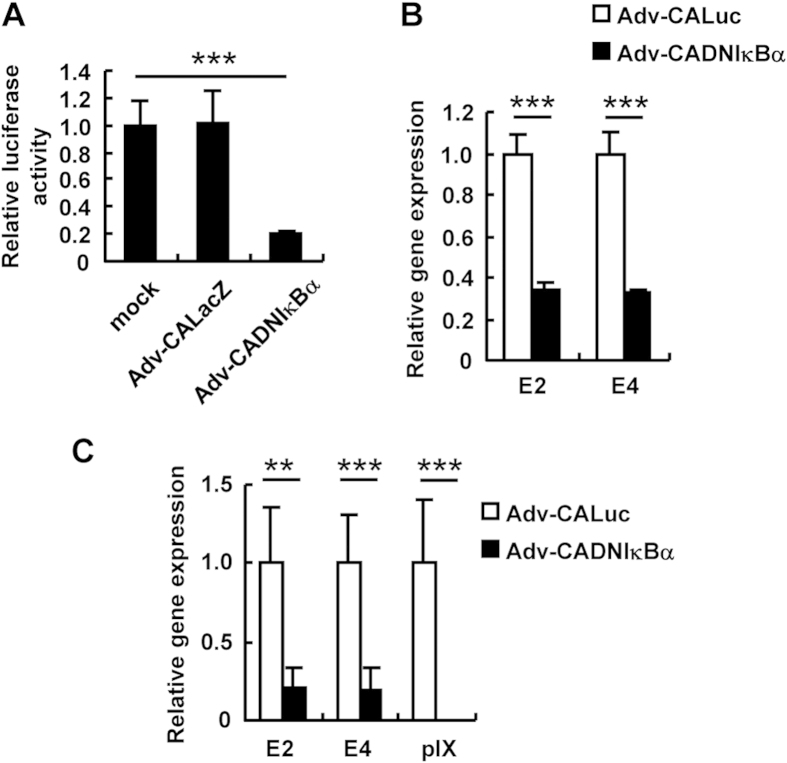
Suppression of Ad early gene expression by expression of a dominant-negative IκBα gene. (**A**) HeLa cells were transfected with pNF-κB-Luc, followed by transduction with Adv-CALuc or Adv-CADNIκBα at an MOI of 5. After 48-h incubation, luciferase activity was determined. The data show FLuc activity normalized by RLuc activity. (**B**) HeLa cells were transduced with Adv-CALuc or Adv-CADNIκBα at an MOI of 5. After 48-h incubation, the E2 and E4 mRNA levels in the cells were determined by quantitative RT-PCR. These data (**A**,**B**) are expressed as the means ± S.D. (n = 4). (**C**) C57BL/6 mice were intravenously administered 10^9^ IFU of Adv-CALuc or Adv-CADNIκBα. Forty-eight hours after the administration, the E2, E4, and pIX mRNA levels in the mouse liver were determined by quantitative RT-PCR. The data are expressed as the means ± S.E. (n = 5–6). **p < 0.01, ***p < 0.001.

Next, in order to examine the effects of NF-κB on the Ad gene expression *in vivo*, mice were intravenously administered WT-Ad and Ad vectors, followed by intravenous injection of recombinant mouse TNF-α (mTNF-α) 24 h after Ad injection. Ad gene expression levels in the liver were determined by quantitative RT-PCR analysis. The liver is a main organ which an Ad vector are distributed to following intravenous administration. Ad gene expression from the WT-Ad genome in the liver increased by more than 9-fold by mTNF-α administration (Fig. 6A). mTNF-α treatment also mediated a significant increase in the leaky expression of the E2 and E4 genes from the Ad vector genome in the liver (Fig. 6B). Next, to inhibit NF-κB signaling in the liver, we constructed an Ad vector expressing a dominant-negative IκBα (Adv-CADNIκBα), which is a negative regulator of NF-κB^26^. Adv-CADNIκBα induced significant inhibition of NF-κB signaling, compared with a conventional Ad vector expressing LacZ (Adv-CALacZ) (Fig. 7A). Compared with the leaky expression levels of the Ad genes from Adv-CALuc, Adv-CADNIκBα mediated an approximately 70% suppression of the leaky expression of the Ad genes in HeLa cells (Fig. 7B) and in the liver (Fig. 7C). These results indicated that NF-κB mediated the leaky expression of the Ad genes following administration with Ad vectors in not only cultured cells but also the organs.

### Inhibition of NF-κB leads to suppression of the leaky expression of Ad genes and reduction in the hepatotoxicities

**Figure 8:**
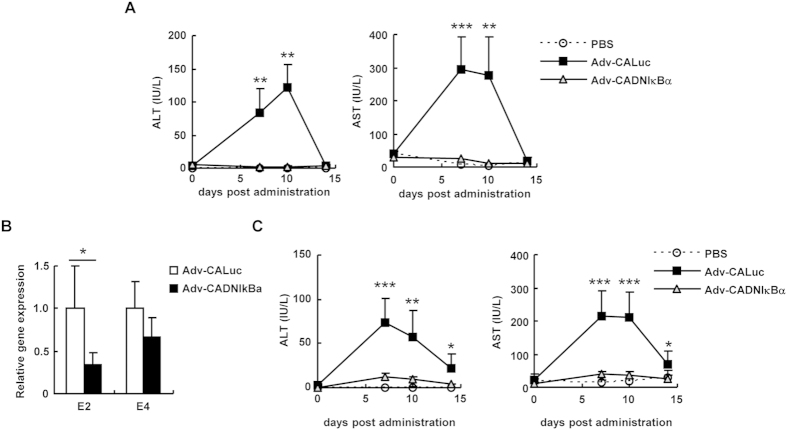
Suppression of Ad vector-mediated hepatotoxicities by expression of a dominant-negative IκBα gene. C57BL/6 mice were intravenously administered 10^9^ IFU of Adv-CALuc or Adv-CADNIκBα. At the indicated time points, the serum ALT and AST levels were determined. These data are expressed as the means ± S.E. (n = 5–6). (**B**,**C**) Rag2/IL2rgc double knockout mice were intravenously administered 10^9^ IFU of Adv-CALuc or Adv-CADNIκBα. (**B**) Forty-eight hours after the administration, the E2 and E4 mRNA levels in the mouse liver were determined by quantitative RT-PCR. These data are expressed as the means ± S.E. (n = 4). (**C**) At the indicated time points, the serum ALT and AST levels were determined. These data (**B**,**C**) are expressed as the means ± S.E. (n = 6–8). *p < 0.05, **p < 0.01, ***p < 0.001 (Adv-CALuc versus Adv-CADNIκBα).

Several groups, including ours, previously reported that the leaky expression of Ad genes from the Ad vector genome induced tissue damages such as hepatotoxicities^7^,^8^,^9^. Therefore, in order to examine whether inhibition of NF-κB leads to the suppression of Ad vector-mediated hepatotoxicities *via* reduction in the leaky expression of Ad genes, serum ALT and AST levels, which are representative biomarkers of hepatotoxicities, were measured at the indicated days after administration with Adv-CALuc and Adv-CADNIκBα (Fig. 8A). ALT and AST levels were not apparently elevated following administration with Adv-CADNIκBα, while Adv-CALuc showed significant elevation in serum ALT and AST levels.

Over-expression of DNIκBα in immune cells might inhibit the activation of immune cells *via* innate immune responses, leading to a reduction in the hepatotoxicities. Ad vector-induced hepatotoxicities are mediated by cytotoxic T cells as well as Ad proteins themselves^7^,^8^,^9^. An Ad vector transduces not only hepatocytes but also dendritic cells, which are professional antigen presenting cells, in the spleen following intravenous administration^27^. Next, in order to examine whether the suppression of Ad vector-mediated hepatotoxicities by DNIκBα expression is induced even in immune-deficient mice, Adv-CALuc and Adv-CADNIκBα were intravenously administered to Rag2/Il2rγ double knockout mice, which have global defects in both cellular and humoral immune responses due to the lack of T, B, and natural killer (NK) cells^17^,^18^. Compared with the leaky expression levels of the E2 genes in the liver of Rag2/Il2rg double knockout mice following intravenous administration with Adv-CALuc, Adv-CADNIκBα exhibited significantly lower expression levels of the E2 genes in the liver (Fig. 8B). Rag2/Il2rγ double knockout mice showed significant elevation in serum ALT and AST levels following intravenous administration with Adv-CALuc, but not with Adv-CADNIκBα (Fig. 8C). These results indicated that NF-κB-mediated leaky expression of Ad genes induced hepatotoxicities, and that Ad-CADNIκBα did not induce any apparent hepatotoxicity due to significant suppression of the leaky expression of Ad genes.

## Discussion

Several studies have demonstrated that the leaky expression of Ad genes from a replication-incompetent Ad vector genome leads to tissue damages such as hepatotoxicities^7^,^8^,^9^; however, the mechanism of the E1A gene-independent expression of Ad genes following transduction has remained unclear. The aim of this study was to identify cellular factors involved in the E1A gene-independent expression of Ad genes and to provide insights into the development of a safer Ad vector. The results showed that NF-κB promoted Ad gene expression following treatment with WT-Ad and an Ad vector (Figs 2, 3, 4 and 6). Adv-CADNIκBα, which efficiently inhibited NF-κB signaling *via* overexpression of DNIκBα, also induced a significant suppression of the leaky expression of Ad genes (Fig. 7). In addition, Adv-CADNIκBα did not lead to any apparent hepatotoxicity following administration (Fig. 8).

This study demonstrated that NF-κB is largely responsible for Ad gene expression. Several types of viruses, including CMV and herpes simplex virus type-1 (HSV-1), utilize NF-κB signaling to facilitate their replication^16^. These viruses possess NF-κB binding sites in their viral gene promoters, and thus the NF-κB signaling triggers the enhancement of their viral gene expressions, leading to promotion of the viral replication. For example, infection with CMV results in up-regulation of Sp1 activity, which induces NF-κB activation. The activated NF-κB triggers the expression of CMV genes, leading to productive CMV infection^28^. HSV-1 also exploits NF-κB signaling for their efficient replication. HSV-1 infection-induced activation of NF-κB significantly promotes the HSV-1 replication *via* up-regulation of expression of the viral late proteins VP16 and gC^29^,^30^. On the other hand, there has been no study reporting the relationship between NF-κB and Ad replication, although Deryckere *et al.* reported that NF-κB up-regulated E3 gene expression. Rather, previous reports demonstrated that the adenoviral E1A^31^ and E3/19K gene products^32^ activated NF-κB in Ad infection. Our present study showed that NF-κB activation enhanced Ad gene expression and Ad replication. These findings suggest that, like other viruses, Ad utilizes NF-κB, which is activated by Ad-mediated innate immunity, for its efficient replication.

Gribaudo *et al.* has previously demonstrated that IFN-α treatment inhibited enhancer activities of murine CMV early genes *via* down-regulation of NF-κB activities^33^. On the other hand, as shown in Figure S3, IFN-α treatment significantly inhibited NF-κB signaling, Ad gene expression, and Ad replication. These results suggest that similarly to murine CMV, Ad infection would be also inhibited by IFN-α signaling *via* down-regulation of NF-κB activities. Further studies will be needed to clarify the mechanism underlying IFN-α-mediated suppression of Ad gene expression.

This study provided evidence that NF-κB is largely involved in not only the gene expression of WT-Ad but also leaky expression of the Ad genes from Ad vectors. Leaky expression of Ad genes is responsible for Ad vector-mediated tissue damages, including hepatotoxicities. Several groups previously reported that Ad vector-mediated hepatotoxicities were significantly reduced by treatment with immunosuppressive agents such as cyclosporine^34^ and FK506^35^, permitting long-term expression of the transgene. In these studies, the diminished hepatotoxicities by the immunosuppressive agents were considered to be mainly attributable to the suppression of Ad vector-induced immune responses; however, immunosuppressive agents also inhibit NF-κB activation, leading to the suppression of leaky expression of Ad genes and the subsequent hepatotoxicities. This study showed that the inhibition of NF-κB *via* overexpression of DNIκBα resulted in a reduction of the Ad vector-mediated hepatotoxicities (Fig. 8).

As shown in Fig. 5A, stimulation by recombinant TNF-α did not activate the E1A, E1B, and E4 promoters, although the E2 and E3 promoter activities were significantly enhanced by TNF-α treatment. The E2 and E3 promoter regions possess the NF-κB binding site (Figs 1B and 5B). On the other hand, *in silico* analysis predicted no NF-κB binding site in the E1A, E1B, and E4 promoter regions, suggesting that NF-κB indirectly induced the enhancement of the E1A, E1B, and E4 gene expressions. Lusky *et al.* reported that the DNA binding protein encoded by the E2A gene is involved in the leaky expression of other Ad genes in Ad vector-transduced cells^4^. It would be interesting to examine whether NF-κB-induced E2 gene products are involved in other Ad gene expression. Alternatively, cellular factors which are activated by NF-κB signaling would be involved in up-regulation of Ad gene expression. Further studies will be needed to clarify the mechanism underlying NF-κB-mediated enhancement of Ad gene expression.

Although we demonstrated that Adv-CADNIκBα achieved a reduction in the expression levels of Ad genes, and thereby induced no evident hepatotoxicity (Fig. 8), previous studies have reported that an Ad vector-mediated expression of IκBα efficiently inhibited NF-κB activities, resulting in significant apoptosis of hepatocytes followed by severe hepatotoxicities^26^,^36^. Because NF-κB functions as an inhibitor of apoptosis^37^, efficient inhibition of NF-κB by overexpression of DNIκBα induced the apoptosis of hepatocytes in these previous reports. In the present study, 10^9^ IFU of Adv-CADNIκBα was intravenously administered to mice, while the previous studies evaluated the hepatotoxicities following administration with Ad vectors expressing IκBα at doses of more than 4 × 10^9^ IFU^26^,^36^. These differences in the injection doses were likely responsible for the differences in hepatotoxicity levels.

In order to suppress or eliminate the leaky expression of Ad genes, various types of replication-incompetent Ad vectors have been developed^3^,^4^,^8^. Following transduction with E2 and/or E4-deleted Ad vectors, the leaky expression of other Ad proteins was significantly suppressed, leading to decreases of cytotoxic T lymphocyte (CTL) responses and hepatotoxicities^3^,^4^,^8^. In addition, an HD-Ad vector, in which almost all viral coding sequences were deleted, has been developed^11^,^12^,^13^. This HD-Ad vector achieved a reduction in inflammatory cytokine production and tissue damages, leading to persistent transgene expression *in vivo*. However, *in vivo* application of HD-Ad vectors has been limited due to the low titers of HD-AD vectors and complicated production method, which requires special packaging cells and a helper virus for the propagation of HD-Ad vectors. As shown in Fig. 8 Adv-CADNIκBα, which was propagated to a high titer in a conventional method, mediated the reduced leaky expression of Ad genes and hepatotoxicities. In addition, Ad vector-induced inflammatory immune responses are suppressed by the overexpression of DNIκBα. An Ad vector carrying an expression cassette of DNIκBα would be a promising framework for safer and more efficient Ad vectors, although we should pay attention to the injection dose, as described above.

In summary, we have demonstrated that NF-κB induces the leaky expression of Ad genes, and that inhibition of NF-κB leads to suppression of Ad vector-mediated hepatotoxicities by not only Ad vector-induced immune responses but also the Ad proteins themselves. This study provides important clues toward the development of promising Ad vectors.

## Additional Information

**How to cite this article**: Machitani, M. *et al.* NF-κB promotes leaky expression of adenovirus genes in a replication-incompetent adenovirus vector. *Sci. Rep.* **6**, 19922; doi: 10.1038/srep19922 (2016).

## Supplementary Material

Supplementary Information
