# Supplementary material for: NF-κB promotes leaky expression of adenovirus genes in a replication-incompetent adenovirus vector
Source: Sci Rep. 2016 Jan 27;6:19922. doi: 10.1038/srep19922 (PMC4728692; doi:10.1038/srep19922)
Supplement: Supplementary Information [file srep19922-s1.pdf]

SUPPLEMENTARY MATERIAL

NF-κB promotes leaky expression of adenovirus genes in a replication-incompetent adenovirus vector

M. Machitani, F. Sakurai, K. Wakabayashi, K. Nakatani, K. Shimizu, M. Tachibana, and H. Mizuguchi

Supplementary Materials and Methods

Cell viability assay

Cells were treated with BAY11-7082 and MG-132. After 24-h incubation, cell viabilities were determined by staining with AlamarBlue (Life Technologies, Carlsbad, CA) according to the manufacturer's instructions. Cell viabilities of DMSO-treated cells were normalized to 100%.

Supplementary Figures

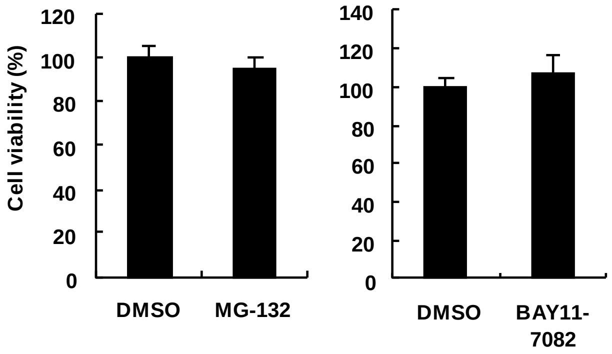

Figure S1 Cell viability following treatment of MG-132 and BAY11-7082.

HeLa cells were treated with MG-132 and BAY11-7082 at 2.5 mM and 10 mM, respectively. After 24-h incubation, cell viability was determined by AlamarBlue assay. Cell viabilities of DMSO-treated cells were normalized to 100%. These data are expressed as the means ± S.D. (n=4).

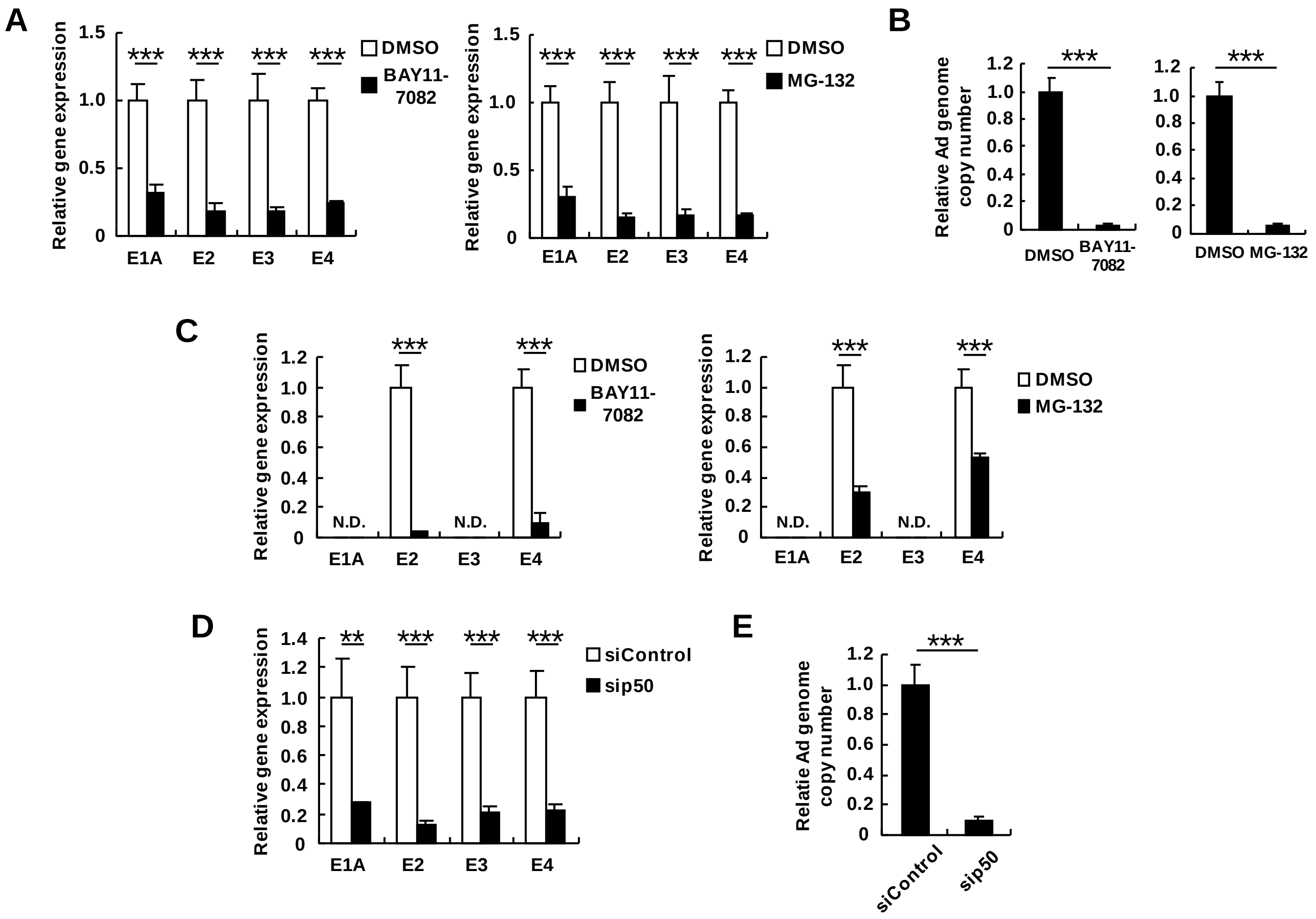

Figure S2 Suppression of Ad early gene expression and Ad replication by inhibition of NF-κB. (A-C) H1299 cells were pre-treated with BAY11-7082 and MG-132 at 10 mM and 2.5 mM, respectively, for 1 h, followed by infection with WT-Ad or Adv-CMVLuc at 100 VP/cell. After 12-h incubation, the E1A, E2, E3, and E4 mRNA levels in the cells were determined by quantitative RT-PCR (A, C). After 24-h incubation, Ad genome copy numbers in the cells were determined by real-time PCR (B). (D, E) H1299 cells were transfected with sip50 at 50 nM, followed by infection with WT-Ad at 100 VP/cell. After 12-h incubation, Ad gene mRNA levels in the cells were similarly determined (D). After 24-h incubation, Ad genome copy numbers in the cells were similarly determined (E). These data are expressed as the means ± S.D. (n=3-4). **p<0.01, ***p<0.001.

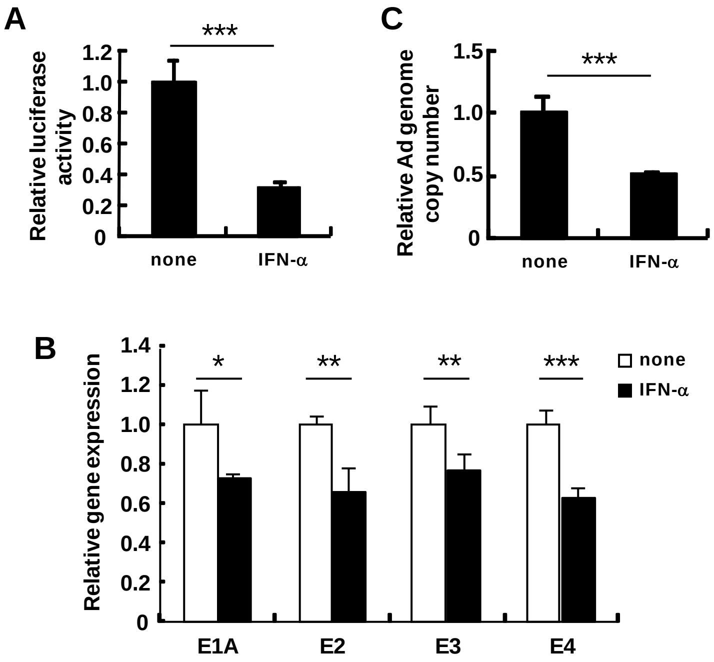

Figure S3 Suppression of Ad early gene expression and replication by IFN-α stimulation.

(A) HeLa cells were transfected with pNF-κB-Luc, followed by treatment with IFN-α at 1000 U/ml. After 24-h incubation, luciferase activity was determined. The data show FLuc activity normalized by RLuc activity. (B) HeLa cells were pre-treated with IFN-α at 1000 U/ml for 5 h, followed by infection with WT-Ad at 100 VP/cell. After 12-h incubation, the E1A, E2, E3, and E4 mRNA levels in the cells were determined by quantitative RT-PCR. (C) HeLa cells were pre-treated with IFN-α at 1000 U/ml for 5 h, followed by infection with WT-Ad at 100 VP/cell. After 24-h incubation, Ad genome copy numbers in the cells were determined by quantitative PCR. These data are expressed as the means ± S.D. (n=3-4). *p<0.05, **p<0.01, ***p<0.001.

Table S1

The oligonucleotides and primers used in this study

No	name	sequence
1	E1Ap-F	atactcgagcatcatcaataatataccttattttggattga
2	E1Ap-R	tataagcttgtcggagcggctcggag
3	E1Bp-F	atactcgaggtgtctagagaatgcaatagtag
4	E1Bp-R	ataaagctttaaccaagattagcccacgg
5	E2p-F	atactcgagtaggattgcctgacgaggcg
6	E2p-R	ataaagctttactgcgcgctgactcttaagg
7	E3p-F	atactcgaggcagctgcctgtatcacaaa
8	E3p-R	ataaagcttagctgaatacctcgccctct
9	E4p-F	gcgaagcttcagtcagccttaccagtaaaaaag
10	E4p-R	gcgctcgagcatcatcaataatataccttattttgg
11	E2-del2-F	atactcgaggctggtaactccacatgtag
12	E2-del2-R	atggtggctttaccaacag
13	E2-del3-S	tcgagctggagatgacgtagttttcgcgcttaaatttgagaaagggcgcgaaactagtcc
14	E2-del3-AS	ttaaggactagtttcgcgccctttctcaaatttaagcgcgaaaactacgtcatctccagc
15	E2-del2.1-F	gtaactccacatgtagggcgtcaattgctcataatggcgctg
16	E2-del2.1-R	cagcgccattatgagcaattgacgccctacatgtggagttac
17	DNIkBa-F	tgtctagacagccatgtttcagccagc
18	DNIkBa-R	aagcggccgcttataatgtcagacgctggcc
19	E1A-F	tccggtccttctaacacacctc
20	E1A-R	acggcaactggtttaatggg
21	E2-F	cactacggtgcgagtgcaa
22	E2-R	ggtagctgccttcccaaaaag
23	E3-F	aacacctggtccactgtcgc
24	E3-R	agctcggagaggttctctcgtag
25	E4-F	gggatcgtctacctccttttga
26	E4-R	gggcagcagcggatgat
27	pIX-F	gcccgcgggattgtg
28	pIX-R	cgggaagctgcactgctt
29	p50-F	aacagagaggatttcgtttccg
30	p50-R	tttgacctgagggtaagacttct
31	hGAPDH-F	ggtggtctcctctgacttcaaca
32	hGAPDH-R	gtggtcgttgagggcaatg

33	mGAPDH-F	caatgtgtccgtcgtggatct
34	mGAPDH-R	gtcctcagtgtagcccaagatg
35	ChIP E2/E3-F	agcgcgaaaactacgtcatc
36	ChIP E2/E3-R	tcccatttgtggctggtaac
